# Suggestions for a New Psychological Terminology

**Published:** 1855-01-01

**Authors:** 


### SUGGESTIONS FOR A NEW PSYCHOLOGICAL TERMINOLOGY.

We are glad to perceive that the "American Association of Medical Officers of Hospitals for the Insane" are taking this subject up in a proper spirit. It is time that all parties connected with the management of institutions for the special treatment of the insane should do their utmost to discountenance the use of terms based upon fallacies, gross, crude, obsolete, and exploded notions, and only calculated to create alarm in the minds of those suffering from brain disorders, and to prejudice and disgust the public against all personally connected with the confinement of the insane. The phrases, "*madness,*" "*mad-house,*" "*lunatic,*" "*lunatic asylum,*" "*keepers,*" "*asylum,*" "*cell,*" should be at once and for ever expunged from our vocabulary. The term "madness" is unquestionably an unscientific one; the word "lunatic" is obviously founded upon an acknowledged error; the appellation "keeper," only suggests to the imagination wild beasts, iron cages, and certain officials who perambulate the Zoological Gardens, and should never escape the lips of humane and scientific men. In fact, it is our duty, recognising the importance of early treatment in cases of insanity, and the necessity, as a curative process, of removing the insane immediately from the excitement and morbid associations of home, to accompany that imperative step with the minimum degree of annoyance, both to the patients and their friends. A man in a state of insanity is not likely to be soothed by being informed that he is going to a "mad-house;" and we would humbly suggest, when there, that the irritation which is necessarily increased by his sudden removal from home, and being placed among strangers, is not likely to be much mitigated by telling him, that he is to be consigned to a "*cell,*" or that he has a "*keeper*" to watch his every movement, to sleep in the same room with him, and, if necessary, to control his actions.

We throw out these *hints* for the immediate consideration of all engaged in the management of this class of patients, intending in an early number to recur to the subject.

